# The Brain-Heart Connection: Frontal Cortex and Left Ventricle Angiotensinase Activities in Control and Captopril-Treated Hypertensive Rats—A Bilateral Study

**DOI:** 10.1155/2013/156179

**Published:** 2013-02-12

**Authors:** Ana B. Segarra, Isabel Prieto, Inmaculada Banegas, Ana B. Villarejo, Rosemary Wangensteen, Marc de Gasparo, Francisco Vives, Manuel Ramírez-Sánchez

**Affiliations:** ^1^Unit of Physiology, University of Jaén, 23071 Jaén, Spain; ^2^Institute of Neuroscience “Federico Oloriz”, University of Granada, 18012 Granada, Spain

## Abstract

The model of *neurovisceral integration* suggests that the frontal cortex (FC) and the cardiovascular function are reciprocally and asymmetrically connected. We analyzed several angiotensinase activities in the heart left ventricle (VT) of control and captopril-treated SHR, and we search for a relationship between these activities and those determined in the left and right FC. Captopril was administered in drinking water for 4 weeks. Samples from the left VT and from the left and right FC were obtained. Soluble and membrane-bound enzymatic activities were measured fluorometrically using arylamides as substrates. The weight of heart significantly decreased after treatment with captopril, mainly, due to the reduction of the left VT weight. In the VT, no differences for soluble activities were observed between control and treated SHR. In contrast, a generalized significant reduction was observed for membrane-bound activities. The most significant correlations between FC and VT were observed in the right FC of the captopril-treated group. The other correlations, right FC versus VT and left FC versus VT in controls and left FC versus VT in the captopril group, were few and low. These results confirm that the connection between FC and cardiovascular system is asymmetrically organized.

## 1. Introduction

Frontal cortex (FC) and cardiovascular functions are reciprocally connected, as part of the model of *neurovisceral integration* [1]. This connection is asymmetric [2] and a neurochemical substrate may underlie this lateralization [3]. Compared with vehicle-treated spontaneously hypertensive rats (SHRs), we recently reported an inverted bilateral behavior of angiotensinase activities between left/right FC and plasma after captopril treatment. The asymmetries between left and right FC markedly increased compared to the control group. We suggested that these results might reflect a systematized lateralized neuroendocrine response between brain and cardiovascular functions involving the autonomic nervous system [4]. There are evidences suggesting that the hyperactivity of the sympathetic nervous system is involved in the cardiac pathologies related to neurological accidents such as cerebral infarction or head traumas, contributing to their high mortality rates [5]. Similarly, it has been also proposed that the autonomic imbalance in which the sympathetic nervous system predominates over the parasympathetic may be the pathway that connects impaired cognitive processes involving frontal cortex functions and altered heart functions [6, 7]. In addition, it was reported that unilateral prefrontal cortex lesions can alter emotional and cardiovascular autonomic responses, depending on which hemisphere was injured: there was a predominant parasympathetic activation by the left prefrontal cortex but a sympathetic inhibition by the right prefrontal cortex [8]. Aspartyl- (AspAP), glutamyl- (GluAP), alanyl- (AlaAP), and cystinyl-aminopeptidase (CysAP) are aminopeptidases (AP) involved in the metabolism of angiotensin peptides [9]. Based on these evidences of neuroendocrine correlations between brain and cardiovascular function and on our previous report showing an asymmetrical effect of captopril between FC and plasma [4], it is therefore essential to analyze those angiotensinase activities in the heart ventricle (VT) of control and captopril-treated hypertensive rats and to search for a possible relationship between these activities in VT and the same determined in the left and right FC.

## 2. Material and Methods

All of the experimental procedures involving animals were performed in accordance with the European Communities Council Directive 86/609/EEC and were approved by the Bioethics Committee of the University of Jaén. Twenty adult male SHRs were divided into control (*n* = 10) and captopril-treated (*n* = 10) groups. Captopril (100 mg/kg p.o.) was administered daily in drinking water (0.5 mL/100 mg body weight) for 4 weeks. The systolic blood pressure (SBP) was monitored by the plethysmographic method throughout the experimental period. At the end of the treatment period, after recording the SBP, each rat was perfused with saline under equithesin anesthesia, and the left and right FC and samples from the left VT were obtained as previously described [4, 10]. Briefly, the brain samples were dissected according to the stereotaxic atlas of Paxinos and Watson [11]. For each group, the left and right frontal lobes 11.20 mm anterior to the interaural line were collected separately [12]. In addition, the heart was removed and weighed and the left ventricle was immediately dissected and weighed and a left ventricular sample was obtained. Soluble (SOL) and membrane-bound (MB) Aspartyl- (AspAP), glutamyl- (GluAP), alanyl- (AlaAP), and cystinyl-aminopeptidase (CysAP) activities were measured fluorometrically using acrylamides as substrates, as previously described [4]. Student's *t*-test was used to compare the data from control and captopril-treated SHRs, and the paired Student's *t*-test was used for left FC versus right FC comparisons [4]. The Pearson correlation coefficient of the left or right frontal cortex and plasma AP activities was computed using SPSS13.0 and STATA 90. *P* values below 0.05 were considered significant. 

## 3. Results

**Figure 1:**
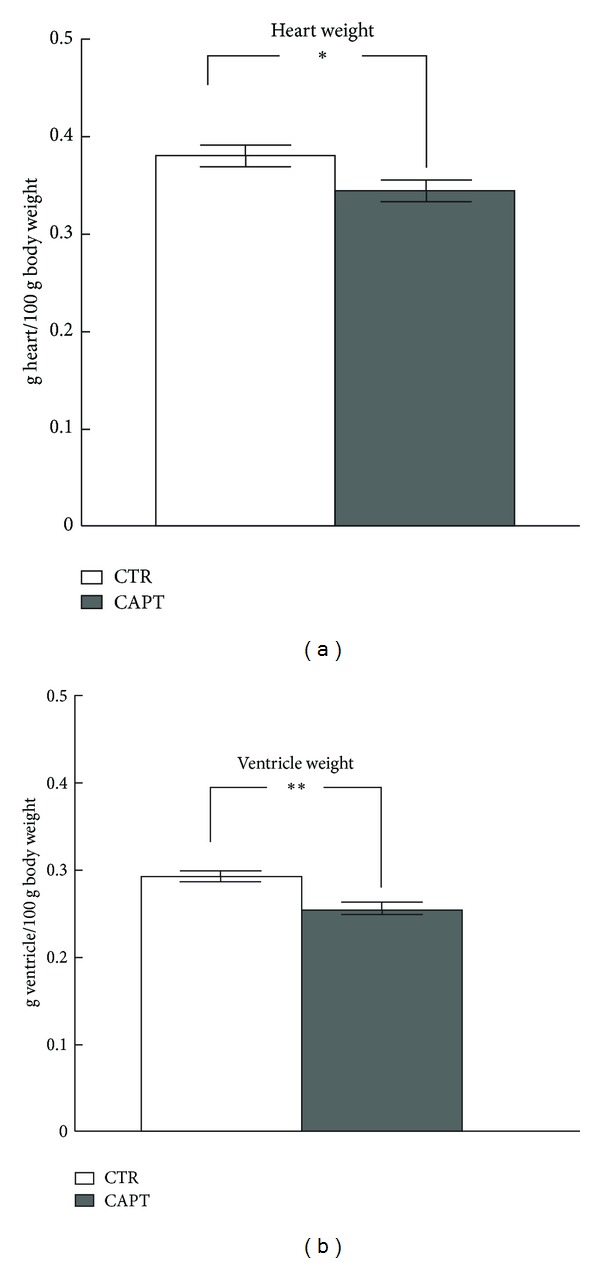
Total heart and left ventricle weight (g) in control (CTR) and captopril- (CAPT-) treated animals, as measured at the end of the treatment period (four weeks). The values represent the mean ± SEM of 10 animals in each group. *P < 0.05; **P < 0.01.

**Figure 2:**
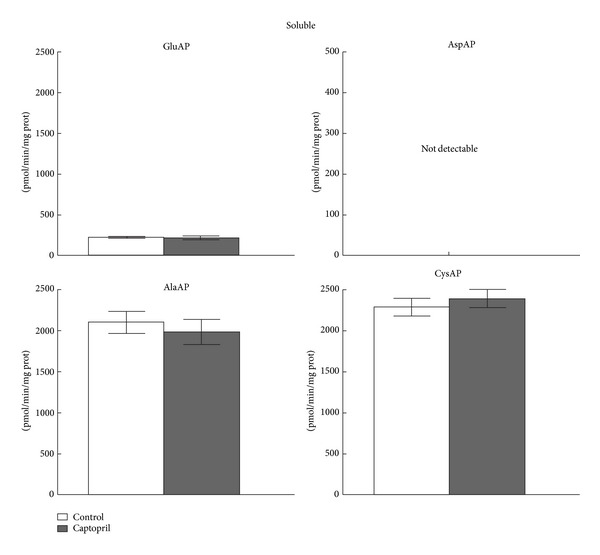
Soluble GluAP, AlaAP, and CysAP activities on the left ventricle of control (*n* = 10) and captopril-treated (*n* = 10) spontaneously hypertensive rats. The values represent the mean ± SEM of specific GluAP, AlaAP, and CysAP activities expressed as picomoles of glutamyl-, alanyl- or cystinyl-*β*-naphthylamide hydrolyzed per min per mg of protein.

**Figure 3:**
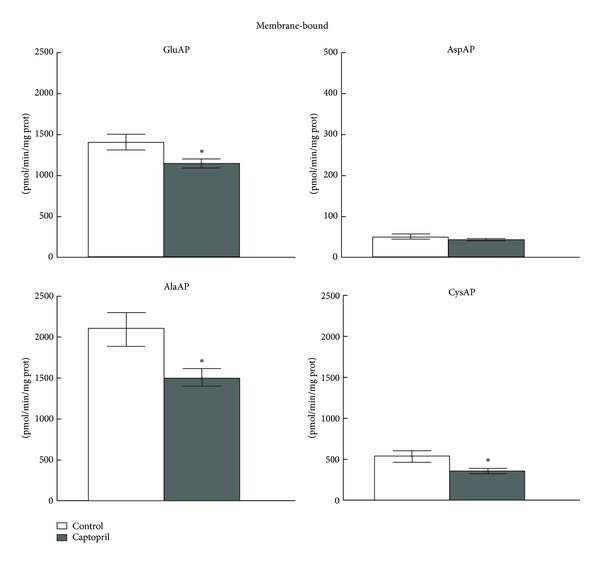
Membrane-bound GluAP, AspAP, AlaAP, and CysAP activities in the left ventricle of control (*n* = 10) and captopril-treated (*n* = 10) spontaneously hypertensive rats. The values represent the mean ± SEM of specific GluAP, AspAP, AlaAP, and CysAP activities expressed as picomoles of glutamyl-, aspartyl-, alanyl-, or cystinyl-*β*-naphthylamide hydrolyzed per min per mg of protein. *P < 0.05.

The results of the present research are reported in Figures 1, 2, and 3 and in Table 1. The SBP of captopril-treated SHRs was 47 mm Hg (or 30%) lower than that of control rats (*P* < 0.001) [4]. The weight of total heart decreased significantly after captopril treatment (*P* < 0.05) mainly due to a reduction in the left ventricle weight (*P* < 0.01) (Figure 1). In a previous study [4], we observed that the asymmetries for MB activities markedly increased in frontal cortex after captopril treatment compared to the control group, whereas the bilateral pattern (left versus right differences) of SOL activities did not substantially change. There was a left predominance for GluAP but a right one for AlaAP and CysAP [4].

In the present study, no differences for SOL GluAP, AlaAP, and CysAP activities were observed between the control and treated SHRs in the ventricle (Figure 2). No detectable activity was measured for SOL AspAP. However, a generalized significant reduction (*P* < 0.05) was observed for all MB AP activities (*P* < 0.05) after captopril except for AspAP that did not reach statistical significance versus the control group (Figure 3). 

There are correlations between FC and VT: the majority and most significant correlations were observed with the right FC of the captopril-treated group. Interestingly, the correlations involving MB activities of the right FC were negative for GluAP (left FC predominant) and positive for AlaAP and CysAP (right FC predominant). The other correlations, right FC versus VT and left FC versus VT in controls and left FC versus VT in the captopril group, were few and low. Surprisingly, the opposite was previously observed between FC and plasma [4]. The plasma AP activities correlated significantly with those in the right FC in the control rats, whereas they correlated with activities in the left FC in the captopril-treated group.

## 4. Discussion

Our results demonstrated that captopril modified angiotensinase activities in heart and in brain, as previously reported [4], and that there is a significant correlation in the levels of these enzymatic activities between both organs.

Several components of a local cardiac RAS can result of an uptake from the circulation but a functional intracrine RAS in the heart also exists [13]. Moreover, ganglionic neurons in human heart express angiotensinogen and are able to generate Ang II [14]. As a whole, the local heart RAS might be involved in cellular hypertrophy and cardiac arrhythmias as well as in the regulation of heart cell volume through the action of Ang II on the AT_1_ receptor [15]. In addition, the presence of Ang IV in the left ventricle was previously described [16]. It was also observed that there was 10-fold more AT_4_ receptor than AT_1_ receptor in rabbit myocardium where they might exert opposite effects to those of Ang II through the binding of Ang IV [16].

The present results of lower MB aminopeptidase activities in ventricle may indicate a local reduction in the metabolism of angiotensin peptides after treatment with captopril. More specifically, the results suggest a lower metabolism/higher availability of Ang II (metabolized to Ang III by GluAP), Ang III (metabolized to Ang IV by AlaAP), and Ang IV (also metabolized by AlaAP) in captopril-treated animals. On the other hand, Ang IV binds specifically to the AT_4_ receptor, which was proposed to be identical to the insulin-regulated aminopeptidase (IRAP) [17]. Indeed, the high affinity-binding site for Ang IV is absent in IRAP-KO mice [18], and the brains of these mice are protected against ischemic damage as Ang IV does in control animals [19]. Cystein aminopeptidase (CysAP), also called oxytocinase or vasopressinase (EC 3.4.11.3), is considered the human variant of IRAP [20]. These enzymes can therefore be considered identical. However, the identity of the AT_4_ receptor remains in dispute, and its identification with IRAP is still controversial. Indeed, Ang IV has a very rapid effect on signaling molecules [21] at pico/nanomolar concentration, whereas the effect of Ang IV on enzyme inhibition such as on accumulation of endogenous IRAP substrates is slow. Also, the concentration of Ang IV producing a biological effect is below that needed to inhibit IRAP [22]. It was therefore proposed that the physiological action of Ang IV was mediated through the tyrosine-kinase cMet receptor whose function overlap with those of Ang IV/AT_4_, that is, memory facilitation, cerebroprotection, seizure, neurite outgrowth, cerebral blood flow, depression, and Parkinson's and Alzheimer's diseases [22].

Independently of the discussion on the real nature of the Ang IV binding site, it was proposed that the binding of Ang IV to its receptor results in the inhibition of the receptor's metabolic activity, reducing the catabolism of its substrates and consequently increasing their availability and extending their action [20]. Ang IV could therefore regulate glucose uptake modulating CysAP activity: CysAP is indeed colocalized with the glucose transporter GLUT4. In the presence of insulin, CysAP and GLUT4 are expressed in the plasma membrane, where GLUT4 induces glucose uptake [20]. Therefore, a decrease in CysAP activity as observed here would imply high levels of Ang IV and increased glucose uptake, which would also improve heart function [23].

These results are in agreement with previous reports indicating that the left ventricular expression of the glucose transporter GLUT4 is regulated by insulin at the transcriptional level [24] and that captopril increased myocardial oxygen and glucose uptake [25]. Therefore, considering these possibilities, the general reduction in the metabolism of Ang peptides in ventricle under captopril treatment might lead to a situation in which Ang peptides counteract each other resulting in an improvement of cardiovascular function.

As previously stated, brain asymmetry is not a static but rather a dynamic phenomenon in which both environmental and endogenous factors act as modulators [3]. In addition, this asymmetry extends to the neurovisceral integration through the asymmetrical functioning of the autonomic nervous system [4, 26–28]. For example, unilateral brain manipulations may not only produce alterations in brain bilaterality [27] but also have significant asymmetrical peripheral consequences in plasma and heart function [26, 28]. In addition, drug treatments may also influence brain bilaterality acting directly and/or indirectly through mechanisms involving the neurovisceral integration model and the asymmetrical functioning of the autonomic nervous system [4]. This hypothesis may agree with the data observed by Chi et al. in 2003 [29] demonstrating that “several genes critical in the establishment of left-right asymmetry were expressed preferentially in venous endothelial cells, suggesting coordination between vascular differentiation and body plan development.” The authors highlighted the importance of the similarity of the migration paths of blood vessels and nerves during development, which reflects their mutual functional interaction [29]. 

It is particularly interesting that the present results are in contrast with those previously observed in plasma [4] where, in the control rats, the plasma AP activities correlated significantly with those in the right FC, whereas they correlated with those in the left FC in the captopril-treated group. These results strongly support that such responses were due to an asymmetry in the organization of the autonomic nervous system's innervations of the blood vessels and heart [4, 26, 28]. Remarkably, the peripheral autonomic innervation not only uses the classic autonomic neurotransmitters acetyl-choline or norepinephrine but also other neuropeptides including angiotensin II that colocalize with them [30]. Thus, neuronal Ang II acts as a neuropeptide and synaptic cotransmitter in the periphery of the sympathetic nervous system [30]. In addition, the present results agree with the notion that essentially the right cortical activity regulates cardiovascular function [2]. Captopril influences heart [31] and brain [32] function and is a clear choice for the treatment of heart failure [33]. In addition, heart function [1] and heart failure [5] affect brain function, and, vice versa, left or right stroke differently influences the heart function [28]. Indeed, the stimulation of the left insular cortex in epileptic patients before temporal lobectomy causes bradycardia and depressor responses, whereas the opposite happens after stimulation of the right cortex indicating a left parasympathetic and a right orthosympathetic dominance, respectively [34]. Cardiac autonomic derangements and arrhythmias have been mainly described in patients suffering right-sided stroke with insular involvement [35]. A reduced respiratory heart rate variability, a reflex mainly under parasympathetic control, was associated with increased mortality after right-sided stroke, suggesting that the risk of sudden death may be correlated with lateralization and the location of the brain infarct after stroke [36]. These and other evidences have led some authors to recommend a prolonged and intensive cardiovascular monitoring in patients manifesting right-sided stroke [37].

There is therefore a clear reciprocal interaction between heart and brain in physiological and pathological conditions [1, 5] that may be modulated by captopril as suggested by the present data. The brain RAS participates in the development of cardiac hypertrophy and fibrosis through the modulation of the autonomic nervous system. Furthermore, the inhibition of the sympathetic hyperactivity after myocardial infarction through suppression of the brain RAS appears to have a beneficial effect [38]. The blood-brain barrier permeability of captopril is negligible, but it has a marked effect on cerebral blood flow autoregulation when injected intravenously [39]. In addition, it causes a sympathetic inhibition in SHRs after chronic administration suggesting that ACE inhibition may be protective for cerebral metabolism against ischemic insult [40]. Therefore, our present results suggest that captopril may centrally modulate the function of the autonomic nervous system leading to a higher connectivity between the right FC and the left ventricle. This could be particularly beneficial after a right-sided stroke. 

The asymmetrical organization of the nervous system is the result of evolutionary adaptation [3, 41]. This is a dynamic phenomenon in which both environmental and endogenous physiological or pathologic factors act as modulators [3]. It has been hypothesized that the brain is intrinsically asymmetric. Virtually all brain functions are organized, more or less, in an asymmetrical fashion. It was speculated that imbalances in established brain asymmetries (toward symmetry or toward increasing asymmetry) might lead to neuropathological deviations in the functions of the nervous system, including the peripheral consequences due to changes in the modulation of the autonomic nervous system [3, 42, 43]. A deeper knowledge of how deviations in physiological lateralizations may lead to neuropathological consequences and how some exogenous factors may influence in physiological laterality, is important in order to be able to search for therapeutic tools that balance those deviations.

Captopril asymmetrically modifies angiotensinase activities in frontal cortex and in ventricle and, consequently, affects the functions they are involved in. However, these effects are not independent but according to our results, mutually interdependent. Therefore, but when appropriately characterized and systematized, the present results open new therapeutic consequences in cardiovascular treatment (presumably not only limited to specific drugs) that should be taken into account in the design of protocols for the evaluation of cardiovascular treatments. The present study provides further molecular support for the involvement of the RAS in the brain frontal cortex-heart connection and adds, for the first time, biochemical evidence on the lateralized functioning of that connection. If this connection is asymmetric and the consequences of unilateral brain insults differ depending on the injured side, the necessity of characterizing appropriately the neurovisceral integration of the RAS is relevant and deserves further preclinical work.

## Figures and Tables

**Table 1:** Frontal cortex versus ventricle.

Left frontal cortex			Right frontal cortex		
FC versus VT	*r*	*P*	FC versus VT	*r*	*P*
Control					
SOL GluAP versus MB CysAP	−0.733	0.03			
MB GluAP versus SOL AlaAP	+0.703	0.05	No correlations		
MB GluAP versus MB GluAP	+0.693	0.05			

Captopril					
MB GluAP versus MB CysAP	+0.766	0.02	SOL CysAP versus SOL AlaAP	+0.700	0.05
			SOL GluAP versus SOL AlaAP	+0.722	0.04
			SOL AlaAP versus MB CysAP	−0.749	0.03
			SOL GluAP versus MB CysAP	−0.781	0.02
			MB CysAP versus MB AlaAP	+0.692	0.05
			MB AlaAP versus MB AspAP	+0.805	0.02
			MB GluAP versus MB AspAP	−0.870	0.01
			MB CysAP versus MB CysAP	+0.695	0.05
			MB AlaAP versus MB GluAP	+0.742	0.03
			MB CysAP versus MB GluAP	+0.707	0.04

Correlations between the left or right frontal cortex (FC) soluble (SOL) or membrane-bound (MB) AP activities versus SOL or MB left ventricle (VT) AP activities in the control and captopril-treated animals. Pearson's correlation coefficients (*r*) and *P* values are indicated and specify the significance of the differences between these correlations.
